# *Salmonella* Bacterin Vaccination Decreases Shedding and Colonization of *Salmonella* Typhimurium in Pigs

**DOI:** 10.3390/microorganisms9061163

**Published:** 2021-05-28

**Authors:** Eduarda Alexandra Gonçalves de Oliveira Moura, Daniela Gomes da Silva, Caio Henrique Turco, Thainara Vitoria Carnevalli Sanches, Gabriel Yuri Storino, Henrique Meiroz de Souza Almeida, Marina Lopes Mechler-Dreibi, Isabela Peixoto Rabelo, Karina Sonalio, Luís Guilherme de Oliveira

**Affiliations:** School of Agricultural and Veterinarian Sciences, São Paulo State University (Unesp), Via de Acesso Prof. Paulo Donato Castellane s/n, Jaboticabal, São Paulo 14884-900, Brazil; eduarda.moura@unesp.br (E.A.G.d.O.M.); danisoulbr@yahoo.com.br (D.G.d.S.); c.turco@unesp.br (C.H.T.); tthainarasanches@gmail.com (T.V.C.S.); gabrielystorino@gmail.com (G.Y.S.); henri_almeida2003@yahoo.com.br (H.M.d.S.A.); mlopesvet@gmail.com (M.L.M.-D.); isabelapeixoto5@gmail.com (I.P.R.); karina.sonalio@unesp.br (K.S.)

**Keywords:** piglets, diarrhea, immunoglobulin, salmonellosis, immunization, inactivated vaccine

## Abstract

Since the occurrence of swine salmonellosis has increased over time and control strategies other than biosecurity are highly recommended, the present study aimed to evaluate the efficacy of vaccination with *Salmonella* Choleraesuis and *Salmonella* Typhimurium bacterins in pigs. Two experimental groups were formed: G1, animals immunized with two doses of a commercial vaccine (*n* = 20); G2, control group (*n* = 20). After vaccination, all pigs were orally challenged (D0) with 10^8^ CFU of *Salmonella* Typhimurium and evaluated for 40 days. Every 10 days after D0, five piglets from each experimental group were euthanized and submitted to the necroscopic examination, when organ samples were collected. Blood samples and rectal swabs were collected before the first dose of the vaccine (D−42), before the second dose (D−21), before the challenge (D0), and thereafter, every three days until D39. Blood count, serum IgG measurement by ELISA, and the excretion of *Salmonella* Typhimurium in feces were evaluated. While the results from blood count and serum IgG concentration did not differ, the detection and excretion of *Salmonella* between G1 and G2 differed (*p* < 0.05). Therefore, it was observed that this vaccine partially protected the animals against experimental infection with *Salmonella* Typhimurium, reducing the excretion of bacteria in feces.

## 1. Introduction

*Salmonella* infection in pigs was first reported in 1886 by Salmon and Smith when *Salmonella* Choleraesuis was associated with hog cholera [1], and since then, more than 2600 serovars of *Salmonella* have been reported, affecting a wide range of animals, including humans [2]. The genus *Salmonella* spp. consists of only two species, *Salmonella enterica* and *Salmonella bongori*, in which *S. enterica* is divided into six subspecies: *S. enterica* subsp. *enterica*, *S. enterica* subsp. *salamae*, *S. enterica* subsp. *arizonae*, *S. enterica* subsp. *diarizonae*, *S. enterica* subsp. *houtenae*, and *S. enterica* subsp. *indica* [1,2]. From the 2637 serovars belonging to the *Salmonella enterica* species, at least 1576 are classified as *Salmonella enterica* subsp. *enterica*, which also includes *S.* Choleraesuis (SC) and *S.* Typhimurium (ST), the most important serovars in pig production worldwide [2,3].

*Salmonella* Typhimurium, a non-host-specific *Salmonella*, recently became the most common serovar isolated from pigs in Europe, the United States, and Brazil [4,5,6,7], and it is one of the main causes of human salmonellosis around the world, characterized by a self-limiting gastroenteritis syndrome, with diarrhea as the main symptom [8,9]. The clinical signs in pigs infected with ST course with the development of enterocolitis, beginning with watery diarrhea (with or without blood) that lasts 3 to 7 days, followed by dehydration and a decrease in feed intake; mortality is usually low [3]. 

On the other hand, *Salmonella* Choleraesuis infection in swine is known for causing generalized sepsis in affected animals, which rarely show signs of diarrhea. At the beginning of the infection, pigs appear lethargic, febrile, and hardly move around the pen, and overall mortality tends to be high [3,10]. Even though clinical signs of swine salmonellosis are usually seen, in many cases, pigs are subclinically infected and therefore serve as a source of infection for healthy animals. In addition to its economic impact on the pig industry, swine salmonellosis plays an important role in public health, since contaminated pork meat is pointed out as the most relevant source of human salmonellosis in several countries [8,9,11,12].

Swine salmonellosis is highly prevalent in the main pork production countries; thus, its control is critical, especially at the herd level [5,13,14,15]. Nearly all on-farm control measures focus on biosecurity, acidification of water and feed, use of probiotics and prebiotics, and vaccination [1,3,13,16]. The farthest measure aims at the prevention of the clinical disease and the decrease of shedding and colonization, which leads to a consequent decrease in carcass contamination at slaughter [17,18,19]. 

Vaccines that stimulate both innate and acquired immunity have a better chance of suppressing the *Salmonella* infection in pigs. It is known that the innate and adaptative immune response against *Salmonella* are crucial for overcoming the infection in the host [20]. While the innate cells play a role during the early stage of an infection, such as controlling bacterial replication and producing cytokines and chemokines that activate and recruit inflammatory cells to the site of infection, the adaptive immunity starts in a later stage of infection via antigen presentation (antigen-presenting cells and dendritic cells) to lymphocytes, aiming at resolving the *Salmonella* infection [1,20,21]. In addition, both the presence of Th1-type immunological memory and anti-*Salmonella* antibodies are necessary to a vaccine-induced resistance, which indicates that the development of resistance against *Samonella* following vaccination depends on the interaction between T and B cells [20,21].

According to de la Cruz et al. [22], most studies on vaccine efficacy are based on the comparison of the presence of *Salmonella* spp. in feces and organs of infected and non-infected pigs; however, the outcome may differ according to the composition of the vaccine in question. Moreover, it was shown that the use of attenuated and inactivated vaccines against *Salmonella* spp. in pigs provide similar estimates of efficacy [22]. Such efficacy confirms why inactivated vaccines have regained some appeal lately, given their safety profile for consumers and zero risks of reversion to virulence. Accordingly, a potential hazard may come with the use of live vaccine strains that, by mutation or acquisition of genetic material in the field, might re-acquire virulent characteristics [17].

Therefore, the present study aimed to assess the efficacy of an inactivated (bacterin) *Salmonella* Choleraesuis and *Salmonella* Typhimurium vaccine in experimentally infected piglets through the detection of *Salmonella* spp. in feces and organs as well as through serological evaluation of immunoglobulin G (IgG) concentration.

## 2. Materials and Methods

### 2.1. Animal Selection

The research work was approved by the Ethics Committee on the Use of Animals (CEUA) of the School of Agricultural and Veterinary Sciences (FCAV/UNESP)-Jaboticabal Campus, under the protocol No 014844/19. For this study, 40 weaned piglets were used, 20 males and 20 females, 21 days old, obtained from a previously selected farm, free of *Salmonella* and with good biosecurity measures and located near the municipality of Jaboticabal-SP.

The animals were transported to the Swine Medicine Laboratory at the FCAV/UNESP-Jaboticabal Campus and housed in collective pens, where they received adequate food for the nutritional requirements of the nursery phase and water ad libitum.

### 2.2. Experimental Design and Sample Collection

The piglets were randomly assigned to two experimental groups, consisting of 20 animals each. The animals of group 1 (G1) were immunized subcutaneously with two doses (21-day interval) of a vaccine against swine pneumoenteritis (commercial formulation), containing strains of *Salmonella* Choleraesuis, *Pasteurella multocida*, and *Salmonella* Typhimurium inactivated by formalin and suspended into fenicated physiological serum, according to the manufacturer’s recommendations. While the animals in G1 received the commercial vaccine, the animals in group 2 (G2) were not immunized, serving as the control group. 

Twenty-one days after the second dose of the vaccine (D0), all 40 animals were orally inoculated with 10^8^ colony forming units (CFU) of *Salmonella* Typhimurium and evaluated for the subsequent 40 days. The inoculum was prepared according to the recommendations of Wood et al. [23] and Oliveira et al. [24], from a sample of *Salmonella* Typhimurium (RL0971/09) originally isolated from swine feces and naturally resistant to nalidixic acid.

The strain used as the inoculum was subjected to the antimicrobial sensitivity test on Mueller–Hinton agar (CM0337, Oxoid, Basingstoke, Hampshire, UK), by the Kirby–Bauer method [25], using commercials discs impregnated with the following antibiotics: gentamicin, florfenicol, cefotaxime, cephalothin, ciprofloxacin, ceftriaxone, polymyxin B, tetracycline, streptomycin, nalidixic acid, ampicillin, chloramphenicol, sulfamethoxazole + trimethoprim, and novobiocin [26]. The antibiogram test was also used to check whether the *Salmonella* excreted by pigs, after oral inoculation, showed the same pattern of sensitivity or resistance to the antibiotics tested with the strain used in the preparation of the inoculum.

The collection of blood samples and rectal swabs was performed before the vaccination, on D–42 and on D–21, and before the inoculation (D0). After that, the samples were collected every three days until the thirty-ninth day after inoculation (D39). Blood samples were collected by the puncture of the jugular vein after antisepsis with 70% alcohol, using the vacuum collection system (BD Vacutainer, Franklin Lakes, NJ, USA), while the fecal samples were collected directly from the rectum of the animals with the aid of sterile swabs and immediately placed into tubes with 10 mL of cystine selenite broth (CS) (CM0699, Oxoid) and incubated for 24 h at 37 °C. Blood samples were collected into sterile tubes containing ethylenediaminetetraacetic acid (EDTA) for performing a blood count and into sterile tubes without anticoagulant for serum IgG measurement. Immediately before sampling, the piglets were weighed and subjected to physical examination [27].

Every 10 days after D0 (D10, D20, D30, and D40), five piglets from each experimental group were euthanized according to the guidelines of the National Council for the Control of Animal Experimentation (CONCEA) and submitted to the necroscopic examination. Samples of lungs, liver, spleen, mesenteric lymph nodes, ileum, cecum, and ileocolic lymph nodes were collected aseptically, placed into sterile plastic bags (Whirl-Pak, B01592WA, Madison, WI, USA), and kept under refrigeration until processing.

### 2.3. Blood Count

For all blood samples collected with EDTA anticoagulant, red blood cell count, leukocyte and platelet counts, globular volume, hemoglobin content, and hematimetric indexes, including mean corpuscular volume (MCV), mean corpuscular hemoglobin (MCH), and mean corpuscular hemoglobin concentration (MCHC), were measured in an automatic device (pocH-100 iV Diff, Sysmex Corporation, Kobe, Japan), following the manufacturer’s instructions. The differential leukocyte count was performed based on blood smear count of 100 cells, stained with modified Rosenfeld stain, under optical microscopy, according to Thrall [28].

### 2.4. Serological Analysis (IgG)

Serum IgG concentration was assessed using a commercial Enzyme-Linked Immunosorbent Assay (ELISA) kit (Pig IgG ELISA kit, E101-104, Bethyl Laboratories, Montgomery, TX, USA), according to the manufacturer’s recommendations, in seven specific moments: D–42, D–21, D0, D9, D18, D30, and D39. All plates were read under 450 nm, in an iMark Microplate Reader (Bio-Rad, Hercules, CA, USA).

### 2.5. Bacteriological Analysis

After fecal collection and the selective enrichment stage, 10 µL of Selenite Cystine (SC) broth (CM0699, Oxoid) was transferred to plates containing modified-brilliant green agar (CM0329, Oxoid) with 50 μg/mL of nalidixic acid and then incubated for 24 h more at 37 °C [24]. Colonies presenting characteristic morphology of the *Salmonella* genus were biochemically tested using triple sugar iron agar (TSI) (CM0277, Oxoid) and lysine iron agar (LIA) (CM0381, Oxoid). Then, slide-agglutination was performed using *Salmonella* polyvalent anti-somatic (anti-O) serum and serogroup B somatic anti-antigen serum (Probac do Brasil, São Paulo, SP, Brazil), which included *S.* Typhimurium.

Organ samples were pre-enriched in 2% buffered water (CM0509, Oxoid) in a 1:10 ratio before the selective enrichment step. The samples were homogenized and incubated for 24 h at 37 °C. Subsequently, 1 mL aliquots were transferred to tubes containing 9 mL of CS broth, incubated, and processed under the same abovementioned conditions.

### 2.6. Statistical Analysis

The results regarding blood count, rectal temperature, body weight, and serum IgG concentrations were subjected to analysis of variance (ANOVA) and Student’s *t*-test for comparison between pairs of means at the level of 5% of significance using the Statistical Analysis System (SAS) program (SAS, 9.1.3 version, SAS Institute, Cary, NC, USA) after assessment of normality by the Shapiro–Wilk test. The variables that did not present a normal distribution were submitted to nonparametric analysis of variance using the Mann–Whitney test for comparison between pairs of means at the level of 5% of significance using the statistical program GraphPad Prism (Version 3.0). The results of microbiological isolation were compared using the chi-square test and, when necessary, Fisher’s exact test was applied [29].

## 3. Results

### 3.1. Physical Examination

The physical examination results of piglets (rectal temperature, consistency of feces, and body weight) are shown in Table 1. Regarding the rectal temperature, a difference was observed between groups only at D6, when the highest value was recorded. 

At this same time, a significant difference was also observed in the percentage of piglets with diarrhea (G1: 35.0% and G2: 65.0%). During the experimental period, 46 episodes of diarrhea were observed in animals from G1 (20.9%; 46/220), with 32 mild cases and 14 moderate cases. In animals from G2, 58 episodes of diarrhea (26.4%; 58/220) were observed, 43 of which were mild, 14 were moderate and 1 was severe. There was no difference between groups (*p* > 0.05). There were also no differences in the body weight of the animals from the two experimental groups over the evaluation period (Table 1).

### 3.2. Erythrogram and Platelet Count

The results of erythrogram (red blood cell count, hemoglobin content, globular volume, MCV, MCH, and MCHC) and platelet count are shown in Table 2. There were no differences in the number of red blood cells, hemoglobin content, and globular volume of animals from the two experimental groups.

Regarding the hematimetric index, significant differences were observed in the MCV and MCH values between groups G1 and G2 at D–42 and in MCHC values at D–42 and D–21. However, these values remained within the range considered normal for swine species. No differences were observed in number of platelets of the animals from two experimental groups (Table S1).

### 3.3. Leukogram

The results of leukocyte count and differential leukocyte count (number of basophils, eosinophils, rod neutrophils, segmented neutrophils, lymphocytes, and monocytes) are detailed in Table S2. Regarding leukocyte count, a difference was observed between groups only in D6, when the highest number of leukocytes was recorded in animals of G2 (24.0 ± 5.04 × 10^3^/µL). Only the animals of G2 showed average values for the number of leukocytes above the range considered normal for swine species.

As for differential leukocyte count, significant differences were observed in lymphocyte count between the two experimental groups at D6 and D12, with higher counts being observed in animals from G2. Significant differences were also observed in the eosinophil count between G1 and G2 groups in D0, in rod neutrophil count in D12, and in monocyte count at D–42 and D3, but all counts remained within the range considered normal for swine species. There were no differences in the number of basophils and segmented neutrophils in animals from the two groups (Table S2).

### 3.4. Serum IgG Concentration

**Figure 1:**
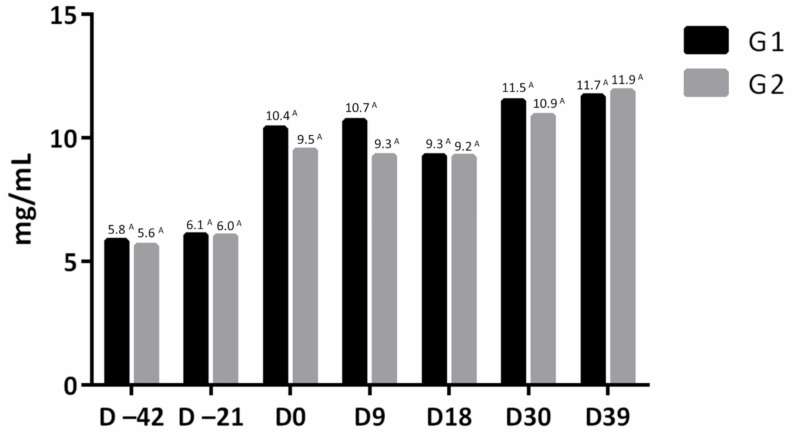
Mean of serum IgG concentration (mg/mL) of immunized (G1) and non-immunized (G2) groups with the inactivated vaccine against swine pneumoenteritis. Means followed by the same letter on the same bar do not differ by Student’s *t*-test (*p* > 0.05).

No difference was observed in serum IgG concentration of immunized (G1) and non-immunized (G2) animals with swine pneumoenteritis vaccine, before the first dose of vaccine (D–42), before the second dose (D–21), before oral inoculation with 10^8^ CFU of *Salmonella* Typhimurium (D0) and at 9 (D9), 30 (D30), and 39 (D39) days after inoculation using the ELISA technique (Figure 1).

### 3.5. Bacteriology and Necroscopic Evaluation

Successful microbiological isolation of *Salmonella* Typhimurium in feces and/or organ samples was possible in all 40 inoculated piglets. In four animals from G1 and two animals from G2, there was no detection of *Salmonella* in feces, but the bacteria were isolated in the organ samples of these animals. On the other hand, although *Salmonella* was detected in the feces of one animal from G2, there was no detection of bacteria in its organs.

Furthermore, the strain used in preparation of the inoculum and *Salmonella* isolated in feces and/or organs of experimentally infected animals showed identical patterns of susceptibility to the tested antimicrobials.

The main necroscopic findings in animals in both groups were the presence of button-shaped ulcers in ileocecal region (G1: 31.6%, 6/19; G2: 35.0%, 7/20) and hyperplasia of lymphoid tissue in colon (G1: 15.8%, 3/19; G2: 25.0%, 5/20) after oral inoculation with 10^8^ CFU of *Salmonella* Typhimurium.

#### *Salmonella* Isolation from Fecal Swabs and Organ Samples

**Figure 2:**
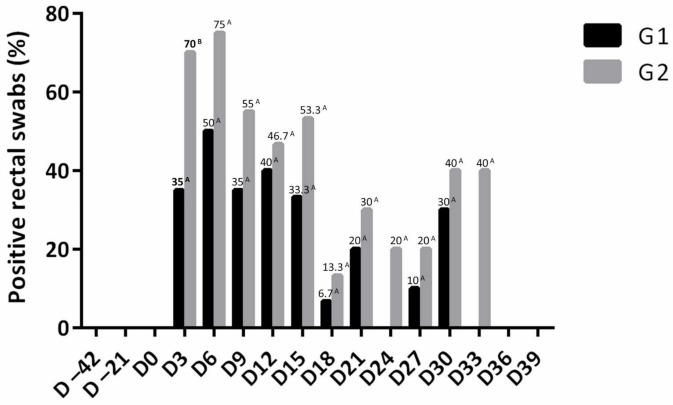
Percentage of rectal swabs positive for *Salmonella* Typhimurium in immunized (G1) and non-immunized (G2) groups with the inactivated vaccine against swine pneumoenteritis. Values followed by different letters on the line differed by the chi-square test or Fisher’s exact test (*p* > 0.05).

Animals from G1 showed lower percentages of positive samples for *Salmonella* Typhimurium during the evaluation period, with a significant difference in D3 (G1: 35.0% and G2: 70.0%). Of the 220 rectal swabs analyzed in each experimental group, it was possible to perform the microbiological isolation of *Salmonella* Typhimurium in 42 samples from animals of G1 (19.1%) and 70 samples from animals of G2 (31.8%), with a significant difference between groups (*p* < 0.05) (Table S3 and Figure 2). It was also observed that from the 6th day after inoculation until the 10th day, most animals were excreting *Salmonella*, suggesting a possible peak of excretion (Figure 2).

**Figure 3:**
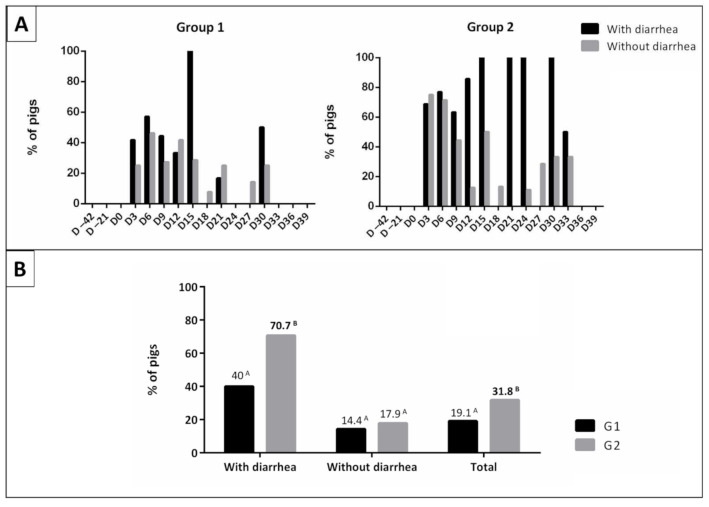
Percentage of positive rectal swabs samples for *Salmonella* Typhimurium, according to the consistency of the feces. (**A**) Vaccinated animals (G1) and non-vaccinated animals (G2). (**B**) Comparison between the averages in pigs with and without diarrhea, and the total. Values followed by the same letter do not differ from each other by the chi-square test (*p* > 0.05).

Regarding animals with diarrhea, microbiological isolation of *Salmonella* Typhimurium was performed in 17 samples from animals of G1 (40.0%; 17/46) and 41 samples from animals of G2 (70.7%; 41/58), with a significant difference between the groups (*p* < 0.05) (Figure 3 and Table S4).

There were no differences in the percentage of organ samples positive for *Salmonella* Typhimurium between the two groups at the evaluated times. Microbiological isolation of *Salmonella* Typhimurium was successful in all evaluated organs, except in the spleen. There were 45 positive samples from animals in G1 (33.8%; 45/133) and 54 positive samples in animals from G2 (Table 2).

## 4. Discussion

As previously stated, the stimulation of immune response by vaccines is a useful mechanism for fighting pathogens, and since it is an important control strategy in countries with a high prevalence of salmonellosis in animals, this study aimed to access the efficacy of an inactivated *Salmonella* Choleraesuis and *Salmonella* Typhimurium vaccine in experimentally infected pigs. We hypothesized that a vaccine combining SC and ST in its formula may decrease *Salmonella* excretion, which was confirmed, along with the decrease in organ colonization by *Salmonella* Typhimurium. Accordingly, Ruggeri et al. [30] suggested that vaccination with an inactivated vaccine of *Salmonella* was able to reduce the prevalence of infection, fecal shedding, and organs colonization in pigs. Moreover, Alborali et al. [31] suggest that it may be possible to develop new effective vaccine strategies for the treatment of swine simultaneously infected by different serovars of *Salmonella*, such as SC and ST, commonly found in the field. 

In this study, although the vaccine did not fully protect the animals from developing clinical disease and tissue invasion, shedding and colonization were reduced in the vaccinated group, which was also reported by Gradassi et al. [32], where *S.* Typhimurium bacterin given orally in two doses did not protect fattening pigs but decreased shedding. Additionally, it was noted that on the sixth day after inoculation, most animals were excreting *Salmonella* spp., suggesting a possible peak of excretion, as described previously [24]. Moreover, our results revealed that not only did the infection result in diarrhea, but it spread beyond the gastroenteric limits since the inoculated bacteria were recovered from other internal organs, such as lung and liver, a fact also reported by Oliveira et al. [24].

Serological measurement of IgG did not differ between the vaccinated and non-vaccinated groups, which could be associated with the cutoff of optical density (OD), the antigens used as coating, and the isotype of immunoglobulin used as the conjugated secondary antibody [33]. Similarly, Schwarz et al. [34] reported that there was no seroconversion after vaccination, which could be related to the somatic antigens used in the test. Thus, it is necessary to consider a more specific ELISA test to obtain a more precise result on serological conversion. Furthermore, humoral immunity to *Salmonella* infections has a limited effect [17] because of the infection cycle, in which the organism may hide inside the cells, protecting itself from antibodies [17]. In this case, cell-mediated immunity, characterized mainly by a T-helper 1 (Th1) lymphocyte profile associated with activation of macrophages and cytotoxic lymphocytes, appears to be a critical part of effective anti-*Salmonella* immunity [35]. Therefore, the use of a vaccine that stimulates humoral and cellular immunity has a better chance against *Salmonella* infection in pigs.

An increase in temperature in response to a bacterial challenge is common since febrile state enhances the bacteria-killing efficacy of the immune system; however, in the present study, none of the animals presented a rectal temperature higher than 39 °C, which is the acceptable temperature for the pig’s age [36]. Additionally, as reported by Sanchez et al. [37], variations in the temperature of *Salmonella*-infected pigs may be associated with stress. Nonetheless, body temperature of most animals may also be influenced by environmental conditions such as room temperature, which in our study ranged from 18.4 °C to 28.6 °C.

Regarding the hematological findings, small numeric variations were reported in this study; however, none of them were of significance once the results were within the normal parameters for swine [38]. Moreover, it is important to consider that nursing piglets undergo rapid growth and substantial immune system changes, which may result in some variation in hematological parameters [3,37,38]. As recently reported by Sanchez et al. [37], small differences in leucocyte concentrations are not likely of biological significance, and most likely, their results were influenced more by the stress associated with handling and less by the challenge itself.

Hence, the results from this study reinforce the importance of *Salmonella* vaccination in pigs, especially regarding its fecal excretion over time. Nonetheless, vaccines against *Salmonella* in swine intend to prevent the colonization and shedding of the pathogen and the development of subclinical or clinical disease [39]; even though studies focusing on the use of live attenuated vaccines have increased, inactivated vaccine studies continue to be important because killed vaccines are easily administered, cheaper to produce and still more secure than live vaccines [40]. Additionally, it is known that these vaccines stimulate several potentially effective mechanisms of protection, mainly due to antigen exposure, such as lipopolysaccharide (LPS), flagella, or fimbriae that may induce at least some degree of T cell response, in addition to humoral response [21]. Therefore, inactivated vaccines, with appropriate administration protocols and adjuvants, have shown protective effects against antigenically similar strains in experimental challenge and field studies [17,41].

Finally, *Salmonella* infections in pigs must be considered of concern for two main reasons: the first is the clinical disease, and the second is the food-borne disease, which represents a threat to human health. Furthermore, given that pork meat is one of the most consumed animal proteins in the world, and that non-typhoid *Salmonella* infection in pigs is the main source of carcass contamination at slaughter, alternatives such as vaccination are increasingly important in controlling the disease and in decreasing excretion of the pathogen along the production chain. 

## 5. Conclusions

*Salmonella* infection in pig production animals is a rising concern worldwide since pork products are commonly associated with human salmonellosis outbreaks. Furthermore, it is known that cross-contamination of pork can be reduced by controlling the disease in pig farms, especially through the use of vaccines. Thus, our results demonstrate that vaccination of pigs with *Salmonella* Choleraesuis and *Salmonella* Typhimurium bacterin partially protected the animals against experimental *Salmonella* Typhimurium infection, reducing the excretion of bacterium in feces and colonization of organs of vaccinated animals. Although the results show some protection against the infection, further studies are needed. Still, our findings should be extrapolated to field conditions with care because there were some limitations regarding the number of animals in the sample and the specificity of serological tests. 

## Figures and Tables

**Table 1:** Mean ± standard deviation of rectal temperature (°C), piglets with diarrhea (%), and weight (Kg) of immunized (G1) and non-immunized (G2) groups with the inactivated vaccine against swine pneumoenteritis.

	Rectal Temperature (°C) ^1^		Pigs Showing Signs of Diarrhea (%) ^2^				Weight (Kg) ^1^	
Moments	Groups		Groups				Groups	
	G1	G2	G1		G2		G1	G2
D–42	38.2 ± 0.34 ^A^	38.1 ± 0.55 ^A^	0.0	(0/20)	0.0	(0/20)	6.43 ± 0.45 ^A^	6.61 ± 1.29 ^A^
D–21	38.9 ± 0.43 ^A^	38.6 ± 0.33 ^A^	5.00 ^A^	(1/20)	0.00 ^A^	(0/20)	10.7 ± 1.51 ^A^	10.9 ± 1.77 ^A^
D0	38.5 ± 0.69 ^A^	38.6 ± 0.57 ^A^	0.0	(0/20)	0.0	(0/20)	17.0 ± 3.14 ^A^	17.5 ± 2.37 ^A^
D3	38.4 ± 0.49 ^A^	38.8 ± 0.72 ^A^	60.0 ^A^	(12/20)	80.0 ^A^	(16/20)	17.8 ± 3.38 ^A^	18.4 ± 3.01 ^A^
D6	38.7 ± 0.30 ^A^	39.3 ± 0.50 ^B^	35.0 ^A^	(7/20)	65.0 ^B^	(13/20)	19.7 ± 3.54 ^A^	20.1 ± 3.35 ^A^
D9	38.7 ± 0.35 ^A^	38.7 ± 0.40 ^A^	45.0 ^A^	(9/20)	55.0 ^A^	(11/20)	21.8 ± 3.94 ^A^	21.8 ± 3.55 ^A^
D12	38.8 ± 0.38 ^A^	38.7 ± 0.29 ^A^	20.0 ^A^	(3/15)	46.7 ^A^	(7/15)	24.5 ± 3.66 ^A^	24.8 ± 3.51 ^A^
D15	38.6 ± 0.24 ^A^	38.7 ± 0.36 ^A^	6.67 ^A^	(1/15)	6.67 ^A^	(1/15)	26.3 ± 3.74 ^A^	26.2 ± 3.82 ^A^
D18	38.9 ± 0.23 ^A^	38.9 ± 0.34 ^A^	13.3 ^A^	(2/15)	0.00 ^A^	(0/15)	27.7 ± 3.60 ^A^	26.7 ± 3.94 ^A^
D21	38.7 ± 0.22 ^A^	39.0 ± 0.17 ^A^	60.0 ^A^	(6/10)	30.0 ^A^	(3/10)	29.0 ± 4.39 ^A^	28.8 ± 3.84 ^A^
D24	38.9 ± 0.38 ^A^	38.8 ± 0.40 ^A^	0.00 ^A^	(0/10)	10.0 ^A^	(1/10)	29.7 ± 5.38 ^A^	29.0 ± 4.02 ^A^
D27	38.1 ± 0.48 ^A^	38.1 ± 0.40 ^A^	30.0 ^A^	(3/10)	30.0 ^A^	(3/10)	30.8 ± 5.37 ^A^	30.4 ± 4.14 ^A^
D30	38.2 ± 0.40 ^A^	38.2 ± 0.37 ^A^	20.0 ^A^	(2/10)	10.0 ^A^	(1/10)	32.3 ± 5.42 ^A^	31.7 ± 4.62 ^A^
D33	38.6 ± 0.47 ^A^	39.0 ± 0.36 ^A^	0.00 ^A^	(0/5)	40.0 ^A^	(2/5)	35.7 ± 7.15 ^A^	34.2 ± 6.43 ^A^
D36	38.2 ± 0.54 ^A^	38.5 ± 0.22 ^A^	0.0	(0/5)	0.0	(0/5)	37.2 ± 7.16 ^A^	35.3 ± 5.65 ^A^
D39	37.9 ± 0.61 ^A^	38.0 ± 0.39 ^A^	0.0	(0/5)	0.0	(0/5)	38.6 ± 7.53 ^A^	36.2 ± 5.87 ^A^

^1^ Means followed by the same letter on the line do not differ by Student’s *t*-test (*p* > 0.05). ^2^ Values followed by the same letter on the line do not differ by the chi-square test or Fisher’s exact test (*p* > 0.05).

**Table 2:** Absolute number and percentage of organ samples positive for *Salmonella* Typhimurium in immunized (G1) and non-immunized (G2) groups with the inactivated vaccine against swine pneumoenteritis at 10, 20, 30, and 40 days after oral inoculation with 10^8^ CFU of *Salmonella* Typhimurium.

Groups	Organ	Timepoint									
		D10		D20		D30		D40		Total	
		+/Total	%	+/Total	%	+/Total	%	+/Total	%	+/Total	%
G1	Lung	2/5	40.0	1/5	20.0	1/5	20.0	0/4	0.0	4/19	21.0
	Liver	0/5	0.0	0/5	0.0	0/5	0.0	0/4	0.0	0/19	0.0
	Spleen	0/5	0.0	0/5	0.0	0/5	0.0	0/4	0.0	0/19	0.0
	Mesentery lymph node	2/5	40.0	0/5	0.0	1/5	20.0	0/4	0.0	3/19	15.8
	Ileum	4/5	80.0	3/5	60.0	3/5	60.0	3/4	75.0	13/19	68.4
	Cecum	5/5	100	5/5	100	5/5	100	3/4	75.0	18/19	94.7
	Ileocolic lymph node	4/5	80.0	1/5	20.0	2/5	40.0	0/4	0.0	7/19	36.8
	**Total**	**17/35**	**48.6 ^A^**	**10/35**	**28.6 ^A^**	**12/35**	**34.3 ^A^**	**6/28**	**21.4**	**45/133**	**33.8 ^A^**
G2	Lung	1/5	20.0	1/5	20.0	2/5	40.0	1/5	20.0	5/20	25.0
	Liver	1/5	20.0	0/5	0.0	0/5	0.0	0/5	0.0	1/20	5.00
	Spleen	0/5	0.0	0/5	0.0	0/5	0.0	0/5	0.0	0/20	0.0
	Mesentery lymph node	4/5	80.0	1/5	20.0	1/5	20.0	0/5	0.0	6/20	30.0
	Ileum	5/5	100	3/5	60.0	4/5	80.0	3/5	60.0	15/20	75.0
	Cecum	5/5	100	5/5	100	5/5	100	4/5	80.0	19/20	95.0
	Ileo-colic lymph node	5/5	100	2/5	40.0	0/5	0.0	1/5	20.0	8/20	40.0
	**Total**	**21/35**	**60.0 ^A^**	**12/35**	**34.3 ^A^**	**12/35**	**34.3 ^A^**	**9/35**	**25.7**	**54/140**	**38.6 ^A^**

Values followed by the same letter on the line do not differ from each other by the chi-square test (*p* > 0.05).

## Data Availability

The data presented in this study are available on request from the corresponding author.

## Supplementary Materials

The following are available online at https://www.mdpi.com/article/10.3390/microorganisms9061163/s1, Table S1: Mean ± standard deviation of the number of red blood cells (x106/µL), hemoglobin content (g/dL), globular volume (%), mean corpuscular volume - MCV (fL), mean corpuscular hemoglobin - MCH (pg), mean corpuscular hemoglobin concentration - MCHC (g/dL), and the number of platelets (x103/µL) of immunized (G1) and non-immunized (G2) groups with an inactivated vaccine against swine pneumoenteritis., Table S2: Mean ± standard deviation of the number of leukocytes (x103/µL), basophils (cells/µL), eosinophils (cells/µL), neutrophil rods (cells/µL), segmented neutrophils (cells/µL), lymphocytes (cells/µL), and monocytes (cells/µL) of immunized (G1) and non-immunized (G2) groups with the inactivated vaccine against swine pneumoenteritis., Table S3: Percentage of rectal swabs positive for Salmonella Typhimurium in immunized (G1) and non-immunized (G2) groups with the inactivated vaccine against swine pneumoenteritis., Table S4: Rectal swabs samples positive for Salmonella Typhimurium, according to the consistency of feces, in immunized (G1) and non-immunized (G2) groups with the inactivated vaccine against swine pneumoenteritis.
