# Supplementary material for: Salmonella Bacterin Vaccination Decreases Shedding and Colonization of Salmonella Typhimurium in Pigs
Source: Microorganisms. 2021 May 28;9(6):1163. doi: 10.3390/microorganisms9061163 (PMC8226585; doi:10.3390/microorganisms9061163)
Supplement: Supplementary file 1 [file microorganisms-09-01163-s001.zip › microorganisms-1226499-supplementary.pdf]

Table S1. Mean ± standard deviation of the number of red blood cells (×106/μL), hemoglobin content (g/dL), globular volume (%), mean corpuscular volume-MCV (fL), mean corpuscular hemoglobin-MCH (pg), mean corpuscular hemoglobin concentration-MCHC (g/dL), and the number of platelets (×103/μL) of immunized (G1) and non-immunized (G2) groups with an inactivated vaccine against swine pneumoenteritis.

	Number of Red Blood Cells ($\times 10^6/\mu L$)[1]		Hemoglobin Content (g/dL) [1]		Globular Volume (%) [1]		MCV (fL) [1]		MCH (pg) [1]		MCHC (g/dL) [1]		Number of Platelets ($\times 10^3/\mu L$) [1]	
**Moments**	**Groups**		**Groups**		**Groups**		**Groups**		**Groups**		**Groups**		**Groups**	
	**G1**	**G2**	**G1**	**G2**	**G1**	**G2**	**G1**	**G2**	**G1**	**G2**	**G1**	**G2**	**G1**	**G2**
**D-42**	6.72 ± 0.43 ^{A}	6.67 ± 0.76 ^{A}	11.8 ± 1.37 ^{A}	12.3 ± 0.90 ^{A}	35.8 ± 3.89 ^{A}	36.7 ± 2.52 ^{A}	53.2 ± 3.81 ^{A}	55.3 ± 3.62 ^{B}	17.5 ± 1.35 ^{A}	18.5 ± 1.27 ^{B}	32.8 ± 0.72 ^{A}	33.3 ± 0.69 ^{B}	776 ± 264 ^{A}	691 ± 225 ^{A}
**D-21**	6.40 ± 0.35 ^{A}	6.38 ± 0.65 ^{A}	10.7 ± 0.44 ^{A}	10.9 ± 0.90 ^{A}	33.3 ± 1.53 ^{A}	33.2 ± 2.38 ^{A}	52.1 ± 1.99 ^{A}	52.2 ± 2.86 ^{A}	16.7 ± 0.71 ^{A}	17.1 ± 1.05 ^{A}	32.1 ± 0.49 ^{A}	32.8 ± 0.87 ^{B}	664 ± 159 ^{A}	603 ± 201 ^{A}
**D0**	7.00 ± 0.50 ^{A}	6.86 ± 0.70 ^{A}	11.7 ± 0.74 ^{A}	11.6 ± 0.95 ^{A}	36.4 ± 2.29 ^{A}	35.8 ± 3.05 ^{A}	52.0 ± 2.26 ^{A}	52.2 ± 2.86 ^{A}	16.7 ± 0.76 ^{A}	17.0 ± 1.04 ^{A}	32.1 ± 0.58 ^{A}	32.5 ± 0.92 ^{A}	612 ± 150 ^{A}	551 ± 131 ^{A}
**D3**	6.97 ± 0.55 ^{A}	6.73 ± 0.75 ^{A}	11.5 ± 0.82 ^{A}	11.2 ± 1.07 ^{A}	35.9 ± 2.58 ^{A}	34.7 ± 3.17 ^{A}	51.6 ± 2.17 ^{A}	51.7 ± 2.87 ^{A}	16.6 ± 0.76 ^{A}	16.7 ± 1.02 ^{A}	32.1 ± 0.45 ^{A}	32.4 ± 0.55 ^{A}	607 ± 150 ^{A}	549 ± 138 ^{A}
**D6**	6.88 ± 0.54 ^{A}	6.82 ± 0.77 ^{A}	11.5 ± 0.84 ^{A}	11.4 ± 1.04 ^{A}	35.6 ± 2.64 ^{A}	35.2 ± 3.12 ^{A}	51.7 ± 2.22 ^{A}	51.8 ± 2.88 ^{A}	16.7 ± 0.80 ^{A}	16.8 ± 1.02 ^{A}	32.3 ± 0.48 ^{A}	32.4 ± 0.60 ^{A}	605 ± 163 ^{A}	609 ± 187 ^{A}
**D9**	6.74 ± 0.49 ^{A}	6.57 ± 0.76 ^{A}	11.5 ± 0.69 ^{A}	11.2 ± 1.23 ^{A}	35.5 ± 2.23 ^{A}	34.4 ± 3.68 ^{A}	52.7 ± 2.17 ^{A}	52.5 ± 2.94 ^{A}	17.0 ± 0.74 ^{A}	17.1 ± 1.02 ^{A}	32.4 ± 0.43 ^{A}	32.6 ± 0.73 ^{A}	548 ± 132 ^{A}	493 ± 147 ^{A}
**D12**	6.67 ± 0.48 ^{A}	6.75 ± 0.58 ^{A}	11.6 ± 0.67 ^{A}	11.8 ± 0.78 ^{A}	35.5 ± 1.97 ^{A}	36.1 ± 2.49 ^{A}	53.4 ± 2.05 ^{A}	53.5 ± 3.06 ^{A}	17.5 ± 0.72 ^{A}	17.5 ± 1.10 ^{A}	32.7 ± 0.38 ^{A}	32.6 ± 0.72 ^{A}	507 ± 116 ^{A}	506 ± 106 ^{A}
**D15**	6.74 ± 0.71 ^{A}	6.71 ± 0.55 ^{A}	11.9 ± 1.23 ^{A}	11.8 ± 0.71 ^{A}	36.2 ± 3.73 ^{A}	35.9 ± 2.33 ^{A}	53.8 ± 2.04 ^{A}	53.6 ± 3.04 ^{A}	17.7 ± 0.76 ^{A}	17.7 ± 1.05 ^{A}	32.9 ± 0.28 ^{A}	33.0 ± 0.42 ^{A}	483 ± 95.0 ^{A}	489 ± 114 ^{A}
**D18**	6.95 ± 0.55 ^{A}	6.73 ± 0.56 ^{A}	12.3 ± 0.58 ^{A}	11.9 ± 0.79 ^{A}	37.2 ± 1.85 ^{A}	36.1 ± 2.43 ^{A}	53.7 ± 2.01 ^{A}	53.7 ± 3.00 ^{A}	17.7 ± 0.80 ^{A}	17.7 ± 1.15 ^{A}	32.9 ± 0.49 ^{A}	33.0 ± 0.66 ^{A}	496 ± 126 ^{A}	454 ± 139 ^{A}
**D21**	6.97 ± 0.59 ^{A}	6.72 ± 0.55 ^{A}	12.3 ± 0.90 ^{A}	11.8 ± 0.97 ^{A}	37.1 ± 2.91 ^{A}	36.0 ± 3.19 ^{A}	53.3 ± 2.26 ^{A}	53.6 ± 2.09 ^{A}	17.6 ± 0.83 ^{A}	17.6 ± 0.66 ^{A}	33.0 ± 0.30 ^{A}	32.9 ± 0.63 ^{A}	512 ± 151 ^{A}	516 ± 154 ^{A}
**D24**	7.08 ± 0.61 ^{A}	7.00 ± 0.48 ^{A}	12.3 ± 0.82 ^{A}	12.2 ± 0.91 ^{A}	38.3 ± 2.71 ^{A}	37.6 ± 2.82 ^{A}	54.3 ± 2.14 ^{A}	53.8 ± 2.34 ^{A}	17.4 ± 0.78 ^{A}	17.5 ± 0.69 ^{A}	32.1 ± 0.50 ^{A}	32.5 ± 0.52 ^{A}	594 ± 160 ^{A}	570 ± 233 ^{A}
**D27**	7.23 ± 0.52 ^{A}	7.02 ± 0.49 ^{A}	12.5 ± 0.66 ^{A}	12.2 ± 0.98 ^{A}	38.5 ± 2.14 ^{A}	37.2 ± 2.91 ^{A}	53.3 ± 2.07 ^{A}	53.0 ± 1.94 ^{A}	17.4 ± 0.82 ^{A}	17.4 ± 0.63 ^{A}	32.6 ± 0.44 ^{A}	32.8 ± 0.50 ^{A}	643 ± 228 ^{A}	645 ± 169 ^{A}
**D30**	6.87 ± 0.60 ^{A}	6.80 ± 0.51 ^{A}	11.9 ± 0.70 ^{A}	11.8 ± 0.87 ^{A}	35.6 ± 2.09 ^{A}	35.2 ± 2.62 ^{A}	52.0 ± 2.02 ^{A}	51.9 ± 2.15 ^{A}	17.3 ± 0.74 ^{A}	17.3 ± 0.61 ^{A}	33.3 ± 0.45 ^{A}	33.4 ± 0.63 ^{A}	603 ± 185 ^{A}	609 ± 163 ^{A}
**D33**	7.07 ± 0.53 ^{A}	6.69 ± 0.68 ^{A}	11.1 ± 0.72 ^{A}	11.5 ± 0.97 ^{A}	37.4 ± 2.22 ^{A}	35.7 ± 2.65 ^{A}	53.0 ± 1.48 ^{A}	53.6 ± 1.84 ^{A}	17.4 ± 0.55 ^{A}	17.3 ± 0.68 ^{A}	32.7 ± 0.13 ^{A}	32.3 ± 0.65 ^{A}	440 ± 37.7 ^{A}	531 ± 116 ^{A}
**D36**	6.87 ± 0.41 ^{A}	6.65 ± 0.64 ^{A}	12.5 ± 0.50 ^{A}	11.0 ± 0.95 ^{A}	35.9 ± 1.45 ^{A}	35.4 ± 2.64 ^{A}	52.3 ± 1.27 ^{A}	53.3 ± 1.78 ^{A}	16.2 ± 0.39 ^{A}	16.5 ± 0.45 ^{A}	31.0 ± 0.33 ^{A}	31.0 ± 0.92 ^{A}	445 ± 82.4 ^{A}	546 ± 106 ^{A}
**D39**	7.26 ± 0.49 ^{A}	6.80 ± 0.65 ^{A}	12.4 ± 0.72 ^{A}	11.8 ± 0.90 ^{A}	37.9 ± 2.20 ^{A}	35.8 ± 2.53 ^{A}	52.3 ± 0.97 ^{A}	52.7 ± 1.87 ^{A}	17.3 ± 0.38 ^{A}	17.4 ± 0.50 ^{A}	33.0 ± 0.36 ^{A}	33.0 ± 0.46 ^{A}	442 ± 82.3 ^{A}	539 ± 125 ^{A}

[1] Means followed by the same letter on the line do not differ by Student's *t*-test ($p > 0.05$).

Table S2. Mean ± standard deviation of the number of leukocytes (x103/µL), basophils (cells/µL), eosinophils (cells/µL), neutrophil rods (cells/µL), segmented neutrophils (cells/µL), lymphocytes (cells/µL), and monocytes (cells/µL) of immunized (G1) and non-immunized (G2) groups with the inactivated vaccine against swine pneumoenteritis.

Moments	Leukocytes ($\times 10^3$/µL) [1] Groups G1	Leukocytes ($\times 10^3$/µL) [1] Groups G2	Basophils (cells/µL) [2] Groups G1	Basophils (cells/µL) [2] Groups G2	Eosinophils (cells/µL) [2] Groups G1	Eosinophils (cells/µL) [2] Groups G2	Neutrophils Sticks (cells/µL) [2] Groups G1	Neutrophils Sticks (cells/µL) [2] Groups G2	Segmented Neutrophils (cells/µL) [1] Groups G1	Segmented Neutrophils (cells/µL) [1] Groups G2	Lymphocytes (cells/µL) [1] Groups G1	Lymphocytes (cells/µL) [1] Groups G2	Monocytes (cells/µL) [2] Groups G1	Monocytes (cells/µL) [2] Groups G2
**D-42**	21.3 ± 5.39 ^{A}	24.5 ± 5.90 ^{A}	0 ± 0	0 ± 0	257 ± 179 ^{A}	428 ± 261 ^{A}	41.7 ± 103	0 ± 0	9.967 ± 4.063 ^{A}	11.753 ± 3.840 ^{A}	10.824 ± 2.210 ^{A}	11.944 ± 2.700 ^{A}	252 ± 106 ^{A}	380 ± 238 ^{B}
**D-21**	19.4 ± 6.45 ^{A}	20.4 ± 4.70 ^{A}	0 ± 0	0 ± 0	128 ± 142 ^{A}	140 ± 179 ^{A}	0 ± 0	0 ± 0	8.656 ± 4.491 ^{A}	8.379 ± 3.005 ^{A}	10.374 ± 2.968 ^{A}	11.028 ± 2.918 ^{A}	231 ± 141 ^{A}	248 ± 107 ^{A}
**D0**	20.5 ± 5.53 ^{A}	20.4 ± 4.47 ^{A}	0 ± 0	0 ± 0	271 ± 255 ^{A}	521 ± 347 ^{B}	13.2 ± 58.8 ^{A}	75.2 ± 179 ^{A}	8.057 ± 4.259 ^{A}	6.975 ± 2.867 ^{A}	11.953 ± 2.983 ^{A}	12.642 ± 2.891 ^{A}	250 ± 84.7 ^{A}	211 ± 135 ^{A}
**D3**	19.8 ± 4.51 ^{A}	22.9 ± 5.61 ^{A}	26.3 ± 86.5	0 ± 0	381 ± 215 ^{A}	364 ± 434 ^{A}	222 ± 190 ^{A}	416 ± 516 ^{A}	6.987 ± 2.614 ^{A}	8.462 ± 4.435 ^{A}	11.638 ± 2.653 ^{A}	13.205 ± 2.770 ^{A}	526 ± 375 ^{A}	413 ± 702 ^{B}
**D6**	18.7 ± 4.13 ^{A}	24.0 ± 5.04 ^{B}	51.9 ± 82.1 ^{A}	47.7 ± 100 ^{A}	320 ± 286 ^{A}	582 ± 656 ^{A}	210 ± 183 ^{A}	416 ± 516 ^{A}	6.348 ± 2.879 ^{A}	8.387 ± 2.773 ^{A}	11.289 ± 2.659 ^{A}	14.139 ± 3.462 ^{B}	485 ± 420 ^{A}	377 ± 366 ^{A}
**D9**	19.3 ± 4.88 ^{A}	22.7 ± 4.10 ^{A}	64.2 ± 107 ^{A}	69.2 ± 128 ^{A}	300 ± 234 ^{A}	510 ± 427 ^{A}	143 ± 184 ^{A}	235 ± 286 ^{A}	8.075 ± 3.733 ^{A}	8.970 ± 2.721 ^{A}	10.522 ± 2.808 ^{A}	12.699 ± 2.506 ^{A}	177 ± 185 ^{A}	192 ± 194 ^{A}
**D12**	17.1 ± 2.44 ^{A}	22.0 ± 6.00 ^{A}	20.2 ± 53.3 ^{A}	27.2 ± 105 ^{A}	324 ± 320 ^{A}	459 ± 349 ^{A}	159 ± 214 ^{A}	384 ± 226 ^{B}	6.529 ± 1.714 ^{A}	7.958 ± 3.812 ^{A}	9.828 ± 1.865 ^{A}	12.662 ± 3.264 ^{B}	261 ± 253 ^{A}	491 ± 365 ^{A}
**D15**	14.7 ± 2.23 ^{A}	18.6 ± 3.37 ^{A}	85.0 ± 138 ^{A}	66.1 ± 117 ^{A}	326 ± 260 ^{A}	362 ± 241 ^{A}	76.3 ± 104 ^{A}	95.7 ± 132 ^{A}	4.450 ± 1.529 ^{A}	6.159 ± 1.893 ^{A}	9.574 ± 1.668 ^{A}	11.587 ± 3.033 ^{A}	178 ± 134 ^{A}	344 ± 289 ^{A}
**D18**	13.9 ± 2.43 ^{A}	17.0 ± 3.40 ^{A}	33.7 ± 58.0 ^{A}	72.9 ± 119 ^{A}	288 ± 186 ^{A}	361 ± 251 ^{A}	86.8 ± 77.4 ^{A}	128 ± 149 ^{A}	3.804 ± 1.373 ^{A}	4.640 ± 1.767 ^{A}	9.474 ± 2.182 ^{A}	11.474 ± 2.303 ^{A}	227 ± 155 ^{A}	278 ± 215 ^{A}
**D21**	13.6 ± 3.34 ^{A}	17.6 ± 4.40 ^{A}	30.5 ± 66.6 ^{A}	12.9 ± 40.8 ^{A}	163 ± 106 ^{A}	338 ± 221 ^{A}	74.1 ± 86,7 ^{A}	389 ± 546 ^{A}	3.896 ± 1.862 ^{A}	5.412 ± 2.459 ^{A}	8.611 ± 1.745 ^{A}	11.139 ± 2.373 ^{A}	186 ± 143 ^{A}	309 ± 302 ^{A}
**D24**	17,5 ± 5,05 ^{A}	20,6 ± 4,41 ^{A}	59,9 ± 80,5 ^{A}	178 ± 172 ^{A}	343 ± 445 ^{A}	448 ± 319 ^{A}	58,8 ± 78,0 ^{A}	92,6 ± 121 ^{A}	4.776 ± 2.460 ^{A}	4.974 ± 2.165 ^{A}	11.541 ± 3.681 ^{A}	14.721 ± 3.409 ^{A}	161 ± 128 ^{A}	216 ± 168 ^{A}
**D27**	17,6 ± 3,47 ^{A}	19,4 ± 4,19 ^{A}	45,8 ± 74,5 ^{A}	66,0 ± 107 ^{A}	310 ± 241 ^{A}	348 ± 234 ^{A}	61,5 ± 101 ^{A}	88.9 ± 160 ^{A}	4.438 ± 1.743 ^{A}	5.207 ± 1.710 ^{A}	11.841 ± 2.588 ^{A}	13.325 ± 3.398 ^{A}	265 ± 181 ^{A}	385 ± 269 ^{A}
**D30**	20.0 ± 4.82 ^{A}	20.7 ± 5.34 ^{A}	120 ± 199 ^{A}	192 ± 273 ^{A}	279 ± 286 ^{A}	273 ± 325 ^{A}	69.4 ± 113 ^{A}	102 ± 135 ^{A}	6.607 ± 3.060 ^{A}	6.791 ± 2.074 ^{A}	11.731 ± 2.773 ^{A}	12.894 ± 3.161 ^{A}	453 ± 241 ^{A}	488 ± 309 ^{A}
**D33**	18.9 ± 5.57 ^{A}	22.2 ± 9.41 ^{A}	143 ± 209 ^{A}	157 ± 215 ^{A}	340 ± 391 ^{A}	331 ± 176 ^{A}	437 ± 398 ^{A}	487 ± 168 ^{A}	9.013 ± 3.024 ^{A}	8.229 ± 3.392 ^{A}	8.611 ± 3.575 ^{A}	7.872 ± 2.667 ^{A}	316 ± 287 ^{A}	283 ± 348 ^{A}
**D36**	18.5 ± 4.39 ^{A}	23.2 ± 6.54 ^{A}	46.4 ± 104 ^{A}	139 ± 196 ^{A}	471 ± 297 ^{A}	313 ± 359 ^{A}	96.6 ± 154 ^{A}	154 ± 247 ^{A}	6.074 ± 2.103 ^{A}	7.055 ± 3.015 ^{A}	11.525 ± 4.353 ^{A}	12.419 ± 5.245 ^{A}	266 ± 322 ^{A}	359 ± 252 ^{A}
**D39**	18.1 ± 3.42 ^{A}	22.2 ± 6.13 ^{A}	69.2 ± 95.9 ^{A}	160 ± 165 ^{A}	422 ± 335 ^{A}	415 ± 251 ^{A}	38.8 ± 86.8 ^{A}	29.2 ± 65.3 ^{A}	5.096 ± 2.597 ^{A}	5.043 ± 2.187 ^{A}	12.170 ± 1.675 ^{A}	13.923 ± 5.539 ^{A}	284 ± 231 ^{A}	268 ± 265 ^{A}

[1] Means followed by the same letter on the line do not differ by Student's *t*-test ($p > 0.05$). [2] Means followed by the same letter on the line do not differ from each other by the Mann-Whitney test ($p > 0.05$).

Table S3. Percentage of rectal swabs positive for *Salmonella* Typhimurium in immunized (G1) and non-immunized (G2) groups with the inactivated vaccine against swine pneumoenteritis.

**Time Point**	**Groups**			
	**G1**		**G2**	
**D-42**	0.0	(0/20)	0.0	(0/20)
**D-21**	0.0	(0/20)	0.0	(0/20)
**D0**	0.0	(0/20)	0.0	(0/20)
**D3**	35.0 ^{A}	(7/20)	70.0 ^{B}	(14/20)
**D6**	50.0 ^{A}	(10/20)	75.0 ^{A}	(15/20)
**D9**	35.0 ^{A}	(7/20)	55.0 ^{A}	(11/20)
**D12**	40.0 ^{A}	(6/15)	46.7 ^{A}	(7/15)
**D15**	33.3 ^{A}	(5/15)	53.3 ^{A}	(8/15)
**D18**	6.67 ^{A}	(1/15)	13.3 ^{A}	(2/15)
**D21**	20.0 ^{A}	(2/10)	30.0 ^{A}	(3/10)
**D24**	0.00 ^{A}	(0/10)	20.0 ^{A}	(2/10)
**D27**	10.0 ^{A}	(1/10)	20.0 ^{A}	(2/10)
**D30**	30.0 ^{A}	(3/10)	40.0 ^{A}	(4/10)
**D33**	0.00 ^{A}	(0/5)	40.0 ^{A}	(2/5)
**D36**	0.0	(0/5)	0.0	(0/5)
**D39**	0.0	(0/5)	0.0	(0/5)

Values followed by the same letter on the line do not differ by the chi-square test or Fisher's exact test ($p > 0.05$).

Table S4. Rectal swabs samples positive for *Salmonella* Typhimurium, according to the consistency of feces, in immunized (G1) and non-immunized (G2) groups with the inactivated vaccine against swine pneumoenteritis.

		With Diarrhea		Without Diarrhea		Total	
	**Timepoint**	**+/Total**	**%**	**+/Total**	**%**	**+/Total**	**%**
G1	**D-42**	0/0	0.0	0/20	0.0	0/20	0.0
	**D-21**	0/1	0.0	0/19	0.0	0/20	0.0
	**D0**	0/0	0.0	0/20	0.0	0/20	0.0
	**D3**	5/12	41.7	2/8	25.0	7/20	35.0
	**D6**	4/7	57.1	6/13	46.2	10/20	50.0
	**D9**	4/9	44.4	3/11	27.3	7/20	35.0
	**D12**	1/3	33.3	5/12	41.7	6/15	40.0
	**D15**	1/1	100	4/14	28.6	5/15	33.3
	**D18**	0/2	0.0	1/13	7.69	1/15	6.67
	**D21**	1/6	16.7	1/4	25.0	2/10	20.0
	**D24**	0/0	0.0	0/10	0.0	0/10	0.0
	**D27**	0/3	0.0	1/7	14.3	1/10	10.0
	**D30**	1/2	50.0	2/8	25.0	3/10	30.0
	**D33**	0/0	0.0	0/5	0.0	0/5	0.0
	**D36**	0/0	0.0	0/5	0.0	0/5	0.0
	**D39**	0/0	0.0	0/5	0.0	0/5	0.0
	**Total**	**17/46**	**40.0** ^{A}	**25/174**	**14.4** ^{A}	**42/220**	**19.1** ^{A}
G2	**D-42**	0/0	0.0	0/20	0.0	0/20	0.0
	**D-21**	0/0	0.0	0/20	0.0	0/20	0.0
	**D0**	0/0	0.0	0/20	0.0	0/20	0.0
	**D3**	11/16	68.8	3/4	75.0	14/20	70.0
	**D6**	10/13	76.9	5/7	71.4	15/20	75.0
	**D9**	7/11	63.3	4/9	44.4	11/20	55.0
	**D12**	6/7	85.7	1/8	12.5	7/15	46.7
	**D15**	1/1	100	7/14	50.0	8/15	53.3
	**D18**	0/0	0.0	2/15	13.3	2/15	13.3
	**D21**	3/3	100	0/7	0.0	3/10	30.0
	**D24**	1/1	100	1/9	11.1	2/10	20.0
	**D27**	0/3	0.0	2/7	28.6	2/10	20.0
	**D30**	1/1	100	3/9	33.3	4/10	40.0
	**D33**	1/2	50.0	1/3	33.3	2/5	40.0
	**D36**	0/0	0.0	0/5	0.0	0/5	0.0
	**D39**	0/0	0.0	0/5	0.0	0/5	0.0
	**Total**	**41/58**	**70.7** ^{B}	**29/162**	**17.9** ^{A}	**70/220**	**31.8** ^{B}

Values followed by the same letter on the line do not differ from each other by the chi-square test ($p > 0.05$)
